# Management of Oro-Antral Communication: A Systemic Review of Diagnostic and Therapeutic Strategies

**DOI:** 10.3390/diagnostics15020194

**Published:** 2025-01-16

**Authors:** Gianna Dipalma, Angelo Michele Inchingolo, Irma Trilli, Laura Ferrante, Angela Di Noia, Elisabetta de Ruvo, Francesco Inchingolo, Antonio Mancini, Stefan Cocis, Andrea Palermo, Alessio Danilo Inchingolo

**Affiliations:** 1 Department of Interdisciplinary Medicine, University of Bari “Aldo Moro”, 70121 Bari, Italy; giannadipalma@tiscali.it (G.D.); angeloinchingolo@gmail.com (A.M.I.); irmatrilli@hotmail.com (I.T.); lauraferrante79@virgilio.it (L.F.); angeladinoia@libero.it (A.D.N.); studio.deruvo@libero.it (E.d.R.); dr.antonio.mancini@gmail.com (A.M.); ad.inchingolo@libero.it (A.D.I.); 2 Maxillo-Facial Surgery, Interdisciplinary Department of Medicine, University of Bari, 70100 Bari, Italy; stefandr.cocis@gmail.com; 3 Department of Experimental Medicine, University of Salento, 73100 Lecce, Italy; andrea.palermo@unisalento.it

**Keywords:** oro-antral communication, oral fistula, oro-maxillary communication, antral communication, sinus communication, sinus fistula, surgical treatment, management

## Abstract

**Aim:** This study aims to evaluate the management of oro-antral communications (OAC) and fistulas (OAF), focusing on treatment strategies based on defect size, epithelialization, and the presence of sinus infections, while exploring both traditional and emerging techniques. **Materials and Methods:** The systematic review was conducted following the PRISMA guidelines and registered on PROSPERO (CDR ID 623251). Using targeted keywords, articles in English published within the last 10 years were analyzed from databases such as PubMed, WoS and Scopus, selecting only clinical studies on human patients. After thorough screening, 20 publications were included in the qualitative analysis, among 734 initially identified. **Results:** Small OACs (<5 mm) were managed conservatively with hemostatic materials, while larger defects (>5 mm) required surgical closure, with the Bichat flap proving highly effective for large defects. Innovative treatments using autologous bone grafts and PRF showed promise in supporting tissue regeneration. In cases with sinusitis, the combination of FESS and intra-oral closure techniques resulted in high success rates for infection resolution and defect closure. **Conclusions:** Treatment outcomes for OAC and OAF are highly dependent on the size of the defect and the presence of sinusitis. Multidisciplinary collaboration, along with timely surgical intervention and adherence to medical therapies, is essential for successful management. Emerging techniques and minimally invasive procedures continue to improve patient outcomes, offering hope for more effective and sustainable solutions in complex cases.

## 1. Introduction

### 1.1. Definition and Main Causes

Oro-antral communication (OAC) is an abnormal connection between the oral cavity and the maxillary sinus, primarily occurring when the thin bony wall separating these structures is perforated [1,2,3,4,5]. This condition often arises during certain dental procedures, such as the extraction of posterior maxillary teeth, or because of trauma, infections, tumors, or other anatomical pathologies [6,7].

### 1.2. Symptoms and Complications

The development of this communication can lead to complications, affecting both the functionality of the maxillary sinus and the oral cavity, potentially resulting in infections and respiratory or oral disturbances [8]. The primary causes of OAC include dental procedures, such as maxillary molar extractions, particularly when the roots are in close proximity to the sinus, which can inadvertently create an opening. Other potential causes encompass dental or sinus infections that compromise the bony structure, facial trauma affecting the floor of the maxillary sinus, and neoplastic conditions that lead to bone erosion [9,10]. Symptoms of OAC vary but commonly include the passage of air or fluids between the mouth and nose, which become particularly noticeable during swallowing [11]. Patients often report pain and discomfort in the affected area, nasal congestion, and sometimes purulent discharge. Additionally, OAC can cause recurrent or chronic sinusitis, associated with pain, fever, and general malaise [12,13].

### 1.3. Diagnosis

Diagnosis of this condition is based on detailed clinical and radiographic evaluations. During an intra-oral examination, the dentist may observe air or fluid passing through the communication and can perform the Valsalva maneuver to confirm its presence [14,15,16,17]. Periapical radiographs may detect bone perforations; however, cone-beam computed tomography (CBCT) is preferred for a more accurate assessment. CBCT offers high-resolution three-dimensional images, making it ideal for evaluating the extent of the communication and the overall health of the maxillary sinus [18,19]. Management of OAC requires specific precautions by both the healthcare provider and the patient to prevent infections, facilitate closure of the communication, and ensure optimal healing [20,21]. In particular, the following are important:

- Age:

This can have a significant impact on the tissue healing process. Oral-antral communication ability (OAC) may be impaired by the reduced regeneration that older patients tend to possess. In younger people, healing is typically faster and surgical treatment outcomes are more predictable.

- Medical comorbidities:

Diabetes mellitus: Patients with diabetes, especially those with uncontrolled diabetes, are more likely to experience complications after surgery, such as infection and delayed healing.

Cardiovascular disease: This may affect the ability to defend against alterations in blood flow and regenerative capacity.

Immunosuppression: Patients with diseases such as HIV and treated with immunosuppressive drugs are at increased risk of infection and surgical errors.

- Fumigator status:

A major risk factor for postoperative complications is smoking. Nicotine and smoking obstruct micro-circulation and prevent ossicles from forming, leading to inadequate or retariated collateral. Smokers have a higher likelihood of postoperative infections and a higher rate of dehiscence in surgical sutures.

Therefore, a preoperative evaluation is necessary to identify the patient’s risk factors through detection and diagnostic testing and to teach the patient about lifestyle changes in order to decide treatment methods in connection to patient factors.

It is evident that choosing the right surgical methods is crucial. Less intrusive methods and treatments that encourage primary healing, like bone grafts or Bichat flaps, may be beneficial for patients who are at greater risk of problems.

The healthcare provider has the responsibility to accurately diagnose OAC, carefully evaluating its size, location, and severity, and utilizing radiographic imaging, such as CBCT, when necessary [22,23]. During the physical examination, the clinician may observe visible signs of infection, swelling, or nasal drainage, which could indicate OAC [24,25]. Additional diagnostic tests, such as the “nasal flow test” or the “water test”, can be used to confirm the communication. These involve introducing a small amount of liquid (e.g., saline) into the oral cavity and observing whether it passes into the maxillary sinus, confirming the presence of OAC [26,27]. Once diagnosed, the healthcare provider must provide the patient with clear and precise instructions to prevent complications and promote healing [28,29]. First, patients should be advised to avoid actions that may increase pressure in the oral and paranasal cavities, such as nose blowing, sneezing with a closed mouth, or engaging in intense physical exertion [30,31,32,33]. These activities can worsen communication and facilitate bacterial entry into the maxillary sinus, increasing the risk of infection [34,35]. Patients should also avoid using straws for drinking, as the suction effect can increase pressure in the OAC area [36]. Vigorous oral rinsing must be avoided to prevent increased intra-oral pressure, which could compromise healing [37]. The healthcare provider will also prescribe antibiotic therapy to prevent infections and recommend decongestants to reduce pressure and improve sinus drainage [38,39,40,41,42]. It is essential that patients adhere strictly to the prescribed regimen, completing the full course of antibiotics and any other recommended therapies [43,44]. Regular monitoring during the healing period is critical: the clinician should assess the progression of OAC closure and decide whether surgical intervention is necessary [45,46]. In cases of infection, such as severe pain, fever, purulent discharge, or swelling, patients should promptly inform the clinician for immediate management [47]. The treatment of OAC depends on its size, duration, and the presence of infection [48]. Small communications, typically 2 mm or less, can often be managed conservatively, as they have a high likelihood of spontaneous closure with conservative measures. These may include sutures to cover communication and, in rare cases, the use of a protective plate [49,50]. Antibiotic support is crucial to prevent infections, along with analgesics for pain control and nasal decongestants to reduce pressure [51,52]. For larger communications (>2 mm) or those that persist and lead to sinus infections, surgical intervention becomes necessary. Surgical treatment options include direct closure with sutures for smaller, recent communications or the use of mucosal flaps, such as the Bichat or Rehrmann flap, to create an effective barrier between the oral cavity and the maxillary sinus [53,54]. When the OAC is larger, persists despite conservative treatment, or becomes infected, surgical closure techniques, such as mucosal grafts or other specialized procedures, may be necessary to seal the sinus and prevent the passage of bacteria and other substances between the oral cavity and the sinus. [55,56]. Patients must strictly adhere to precautions, including avoiding any activities that may generate pressure in the maxillary sinus area, such as nose blowing, using straws, sneezing with a closed mouth, or engaging in activities that increase sinus pressure [57]. Smoking and alcohol consumption should be avoided, as they can hinder healing and increase the risk of infections [58,59]. Oral hygiene should be diligently maintained, following the dentist’s instructions, while avoiding vigorous rinsing to prevent pressure that could interfere with OAC healing. Adherence to prescribed pharmacological treatments, including antibiotics, analgesics, and decongestants, is crucial to reduce the risk of infection and alleviate symptoms [60,61]. Management of OAC requires close collaboration between healthcare providers and patients [62,63]. Clinicians must ensure accurate diagnosis, appropriate treatment, and clear instructions, while patients must diligently follow these recommendations to avoid worsening the condition and facilitate complete closure of the communication [64]. In cases of complications, such as acute or chronic sinusitis caused by OAC, endoscopic maxillary sinus surgery may be necessary in conjunction with intra-oral closure techniques [65]. This type of surgery allows for the removal of secretions or infected material from the maxillary sinus, preventing the progression of infection and facilitating healing [66,67,68]. Endoscopic surgery also improves sinus drainage and ventilation, enhancing the overall success of the treatment [69,70,71,72]. The integration of endoscopic and oral surgical techniques is particularly advantageous in managing complex cases of OAC with sinusitis resistant to antibiotic therapy alone [73,74].

### 1.4. Clinical Management

While research has advanced the management of OAC and oro-antral fistula (OAF), there are still areas of uncertainty, especially regarding comparisons between surgical techniques, the use of innovative materials, and long-term outcome analysis. Further studies are needed to clarify these aspects.

## 2. Materials and Methods

### 2.1. Protocol and Registration

The Preferred Reporting Items for Systematic Reviews and Meta-Analysis (PRISMA) were followed in the conduct of this systematic review, which was then submitted to PROSPERO under the ID of CDR 623251.

### 2.2. Search Processing

Using the keywords “(oro AND antral OR maxillary AND sinus) AND (communication OR fistula) AND (surgical AND treatment OR management)”, we searched databases as Scopus, Web of Science (WoS), and PubMed to find papers relevant to this topic. The search was limited to the last 10 years and only English articles were included. The reviewers, in a double-blind manner, included papers that satisfied the following criteria for inclusion: (1) articles including humans; (2) clinical studies or case series or randomized controlled trials. Exclusion criteria were represented by reviews (systematic and/or narrative) with/without meta-analyses, studies regarding animal models, in vitro studies, non-English studies, and articles without free full text.

### 2.3. Data Processing

After the exclusion of any publications that did not fit the themes examined, the complete texts of the publications that had been included earlier were read as part of the screening process, which involved reviewing the article titles and abstracts selected in the previous identification step. A third reviewer (FI) was consulted in cases of dispute after the reviewers had discussed the chosen articles.

### 2.4. Quality Assessment

The quality of the included papers was assessed by two reviewers, L.F. and I.T., using ROBINS, a tool developed to assess the risk of bias in the results of non-randomized studies that compare the health effects of two or more interventions. Seven points were evaluated, and each was assigned a degree of bias. A third reviewer (F.I.) was consulted in the event of a disagreement until an agreement was reached.

## 3. Results

**Figure 1:**
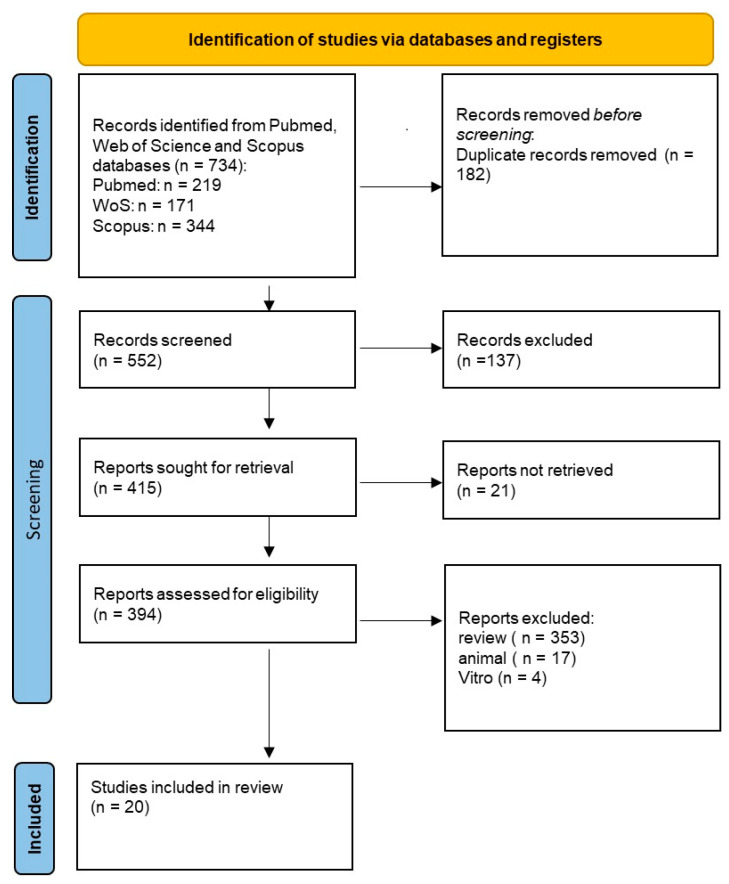
PRISMA flowchart.

734 publications were found after searching three databases: PubMed (219), Web of Science (171), and Scopus (344). Following the elimination of duplicate entries (182), 552 records underwent title and abstract screening, which resulted in the rejection of 137 articles. After a full-text examination, 395 were excluded for not meeting the inclusion requirements. Ultimately, a total of 20 publications were deemed eligible for qualitative analysis (Table 1). The included studies employed a variety of methodologies, e.g., retrospective studies, prospective clinical trials, and observational designs. Most studies focused on evaluating the effectiveness of surgical techniques for repairing oro-antral communications and fistulas, with primary outcomes including the success of defect closure, complication rates, and patient satisfaction. Secondary outcomes, such as pain, swelling, and postoperative recovery times, were also commonly reported. While many studies utilized advanced techniques, such as double-layer closures or the use of bioactive materials, like PRF membranes, others explored non-surgical approaches or novel materials, like 3D-printed meshes. The heterogeneity in study design, sample sizes, and follow-up duration highlights the diversity of the evidence base and underscores the importance of interpreting results within the context of each study’s specific methodology. Despite the heterogeneity among the included studies, we conducted a qualitative synthesis to identify common trends and insights. While this approach captures valuable information, the variability in study methodologies and outcomes highlights the need for caution when extrapolating findings to broader populations. The different methodologies, sample sizes, and follow-up durations of the included studies were considered during data synthesis. Given this variability, a qualitative approach was employed to summarize key findings and trends. Figure 1 provides an overview of the selected procedure.

### 3.1. Quality Assessment and Risk of Bias of Included Articles

**Figure 2:**
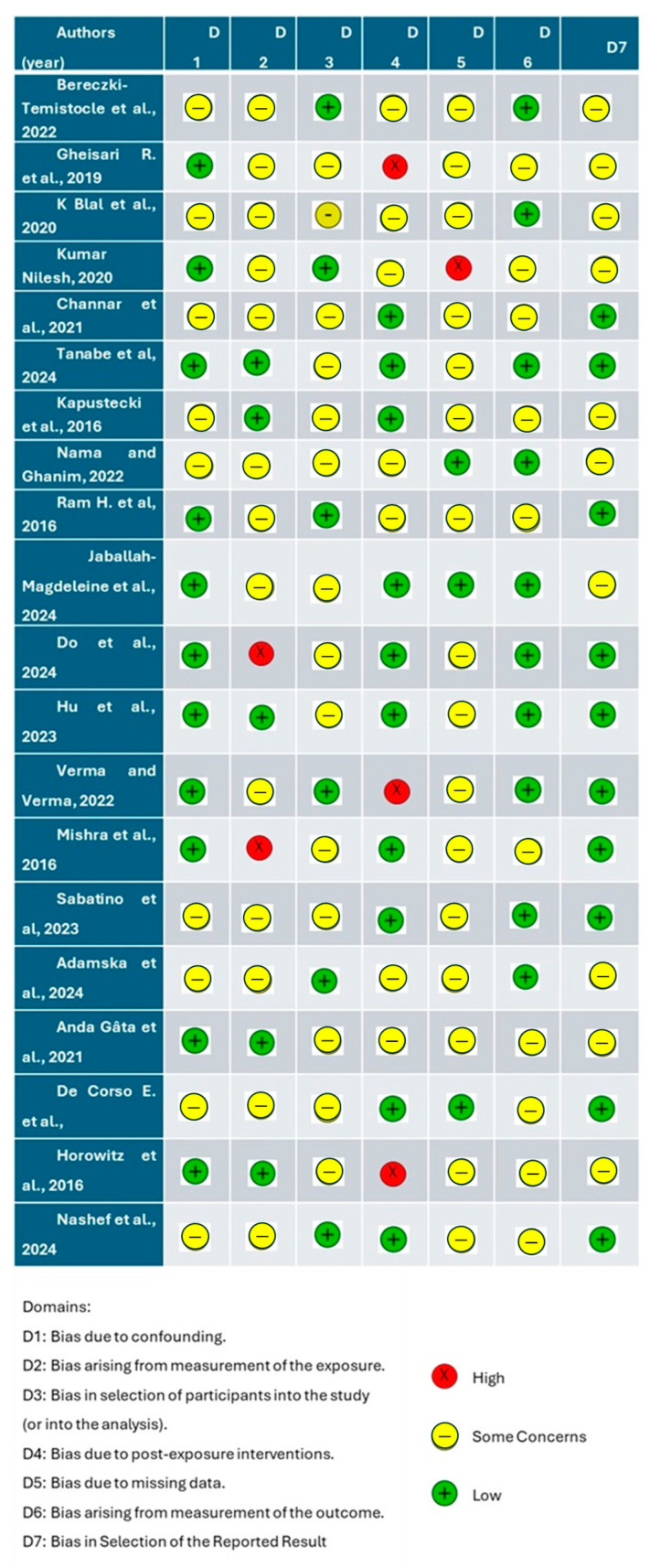
Risk of bias [26,69,75,76,77,78,79,80,81,82,83,84,85,86,87,88,89,90,91,92].

The risk of bias in the included studies is reported in Figure 2. Several studies, such as those by Do et al. (2024) and Horowitz et al. (2016), have been identified as having multiple concerns, particularly in the areas of participant selection, exposure measurement, and handling of missing data [85,91]. These issues can compromise the reliability of their findings, as they may introduce biases that affect the interpretation of the results. On the other hand, many of the studies show a low risk of bias across most domains, especially in post-exposure interventions and the measurement of outcomes. This suggests that these studies adhered to a high standard of methodological rigor. However, even among those studies with generally low risk, some still show concerns in areas like confounding and exposure measurement, indicating that, while the overall methodology is solid, there are aspects that could benefit from improvement. While the risk of bias has been assessed across specific domains, an overall assessment of bias for each study was not explicitly calculated due to the variability in the domains evaluated and the subjectivity inherent in aggregating these into a single score. Providing a comprehensive, domain-specific evaluation allows for a more nuanced understanding of where methodological limitations may influence the reliability of findings. To mitigate this limitation, the narrative synthesis provided in this section emphasizes key domains with significant bias risk and highlights their potential implications on the results. For example, studies with high concerns in participant selection or exposure measurement are discussed in detail to underline their specific limitations. Future research could benefit from standardized tools that combine domain-specific assessments into an overall bias score, though this must be done carefully to preserve the granularity of the domain-specific evaluations. Meanwhile, readers are encouraged to interpret findings with consideration of both the strengths and limitations identified in the domain-level risk assessments provided.

### 3.2. Diagnostic Methods of OAC and OAF

Diagnosis of OAC and OAF includes endoral examination, extra-oral examination, instrumental examination, and symptom analysis.

In the endoral examination, the maneuvers to be performed are inspection, aspiration, irrigation, and the Valsalva maneuver. Inspection with a dental mirror can be useful for large communications, while small ones may not be visible. The suction technique consists of placing the tip of a suction cannula at the communication; in the presence of OAC or OAF, a dull, amplified rumor is felt due to the flow of air generated within the sinus. Irrigation of the defect will be felt by the patient with the passage of fluid toward the nose. The Valsalva maneuver results in an increase in endo-sinusal air pressure so, in the case of OAC or OAF, bubbling, hematoma, seroma, or purulent material may be manifested at the level of the continuous solution between the oral cavity and the sinus.

Extra-oral examination may not reveal anything abnormal in the absence of sinusitis. In the case of sinusitis, swelling, pain and redness of the paranasal region and/or the cheek may be manifested.

To make a definitive diagnosis of OAC or OAF, an endoral or panoramic radiograph is taken using a probe or guttapercha cone inserted into the communication or fistula. In the case of sinus infection, computed tomography is indicated.

The symptomatology of OAC or OAF is highly variable; typically, the patient manifests a sensation of air or fluid passing between the oral and nasal cavities in the absence of pain. Acute inflammation of the paranasal sinus mucosa may cause pain that is aggravated by palpation of the anterior paranasal sinus wall.

## 4. Discussion

Despite the heterogeneity among the included studies, we conducted a qualitative synthesis to identify common trends and insights. While this approach captures valuable information, the variability in study methodologies and outcomes highlights the need for caution when extrapolating findings to broader populations. The maxillary sinus, the largest paranasal cavity, undergoes a process of progressive expansion towards the alveolar process throughout life [93,94]. As a result, this anatomical situation can put one at risk of creating a OAC during oral surgeries involving the posterior region of the upper maxilla. The appropriate treatment of a OAC is necessary to avoid the occurrence of infection in the maxillary sinus [95,96].

Histologically, an OAC must be distinguished from an OAF, but in fact the clinic always performs a clinical diagnosis. An OAC is characterized by the presence of a connection between the oral cavity and the maxillary sinus without an epithelial lining. The OAC may heal spontaneously; in fact, they represent an early stage of the pathological process [97,98]. The OAF is an epithelium-covered via, which represents a more advanced stage of the pathological process. Epithelialization of the via, which occurs within 24–48 h, prevents spontaneous healing of the communication between the oral cavity and the maxillary sinus [72,99,100].

The type of treatment to be performed requires the evaluation of three parameters: the width of the communication, the epithelialization or not of the communication, and the presence or absence of sinus infection [101,102]. The extent of communication is determined by using a probe to measure the diameter of the defect.

In the case of a small OAC (<5 mm) and in the absence of sinus infection, it is possible that the primary clot formed in the postoperative period will lead to spontaneous healing. The healing process can be facilitated by using hemostatic materials, such as oxidized cellulose and fibrin sponges, and by advising the patient to avoid maneuvers that may cause increased sinus pressure [103,104,105].

In the presence of a large OAC or OAF and in the absence of sinus infection, surgical closure of the via is indicated with a local rotational or sliding flap. The most commonly used local flaps are the vestibular flap, the palatine flap, and the Bichat flap. Placement of these flaps requires good vascularization and the ability to close the communication without tension to achieve healing by first intention [106,107].

The vestibular is a full-thickness trapezoidal flap of the random superior pedicle type. Adequate mobilization of the flap through a periosteal release incision is necessary to close the communication [108,109].

The palatal flap is created through a full-thickness incision of the palatal fibro-mucosa to obtain a posterior-based axial pedicle supplied by the greater palatine artery. The flap is fully dissected from the bony plane, rotated, and positioned to cover the communication. At the donor site, healing occurs by secondary intention [110,111].

In the fat bladder, a vestibular flap is created along the edges of the communication, the box containing the bladder is exposed through a periosteal incision, and the bladder is released by blunt dissection with scissors, leaving it pedicled [112,113]. The bladder is sutured at the communication margins and the vestibular flap is then repositioned without the need for periosteal release over the bladder. The large amount of adipose tissue available allows the closure of very large lesions [114,115,116].

If a sinus infection is present, surgical closure of the via must be preceded by resolution of the infection to prevent sinus empyema from draining. To treat the sinus infection, sinus rinses through the via are indicated with an antibiotic solution for approximately one week and, in more severe cases, this should be combined with systemic antibiotic therapy [17,117,118].

For chronic sinusitis that does not resolve with local lavage and systemic antibiotic therapy, a trans-nasal functional endoscopic sinus surgery (FESS) approach is indicated. FESS is a minimally invasive procedure that enables the removal of infected material from the paranasal sinuses using endoscopic instruments to dilate the natural sinus ostium. Closure of the oro-antral communication is performed during the same surgical session using the intra-oral techniques described above [119,120,121].

In the past, chronic sinusitis was treated with the Caldwell–Luc technique, which was too invasive, not always effective, and carried the risk of scarring and inadequate resumption of sinus ventilation. The Caldwell–Luc technique involved a wide intra-oral opening of the anterolateral sinus wall, a nasal counter-opening in the inferior meatus, and removal of a large portion of Schneider’s membrane [122,123].

### 4.1. Small OAC in the Absence of Sinusitis—Clinical Management

The OAC is a non-epithelial conduit that connects the oral cavity with the antral sinus. The lack of epithelialization of the communication, which indicates an early stage of the pathological process, ensures the possibility of spontaneous healing of the OAC.

In the absence of sinus infection, a small OAC (<5 mm) may spontaneously resolve by means of the primary clot formed during the healing period. This process of spontaneous resolution of a small OAC can be clinically facilitated by the use of hemostatic materials, such as oxidized cellulose and fibrin sponges. In addition, it is critical to instruct the patient to avoid maneuvers that may cause increased sinus pressure.

### 4.2. Large OAC or OAF in the Absence of Sinusitis—Traditional Techniques

In the surgical treatment of large OAC or OAF in the absence of sinus infection, the traditional approach is to create local rotational or sliding flaps.

Several authors have conducted studies to identify the most effective surgical technique. Bereczki-Temistocle et al. performed a retrospective study analyzing the medical records of 140 patients with OAC, treated with the buccal advancement flap, the fat bubble flap (Bichat), or the palatal flap. The Bichat flap has demonstrated a 100% success rate and is therefore recommended for larger defects (0.6–1.5 cm) and for recurrences, which are particularly common in patients who have undergone radiotherapy or those with compromised health (e.g., diabetes, smoking, cardiovascular disease). In contrast, the buccal advancement flap has a higher recurrence rate (25% of patients experienced dehiscence) in larger defects, making it more suitable for small to medium-sized defects [75,124].

The experimental evidence that the Bichat flap is the most appropriate for the treatment of OAC has been confirmed by other studies in the literature, including the following by Gheisari et al. The authors compared the efficacy of the same types of surgical flaps, namely the buccal flap (Rehrmann technique), the Bichat flap, and the palatal flap (rotational advancement), for the treatment of OAF. Like the previous study, the Bichat flap had the highest success rate, confirming that it is the most suitable for the treatment of large OAF (>5 mm). In contrast, the palatal flap proved to be the best for medium and small OAF, while the buccal flap showed the least effectiveness compared to the other two [76,125].

Blal et al. evaluated the efficacy of closure of an OAF using a pedicled palatal periosteal flap and concluded that it has a good success rate and can promote gingival tissue regeneration over the bone defect [77].

Other authors have conducted studies to analyze whether it is more effective to treat OAC or OAF with a double flap surgical technique than with the traditional method of treatment using a single surgical flap. Most of the studies showed that the surgical technique of closing OAC with two layers, although more time-consuming and invasive, was more effective and safer than the treatment method with a single surgical flap [17,126].

Kumar Nilesh conducted a randomized, prospective, double-blind clinical trial to compare the closure of an OAC using a two-layer technique (Bichat flap and buccal flap) versus a one-layer technique (Bichat flap alone). The study evaluated several parameters, including complete closure of the communication (surgical success), postoperative pain, swelling, and mouth opening. Although the two-layer technique took more time, it proved to be the most effective, achieving complete closure in all patients. No statistically significant differences were observed in postoperative pain, swelling, or mouth opening between the two groups [78,127,128].

Channar et al. conducted a study like the previous one, comparing closure of an OAF with two layers, i.e., Bichat flap and buccal flap, and closure of an OAF with only one layer, i.e., the buccal flap. The two-layer closure technique was slightly more effective than the other, but this difference was not found to be statistically significant [79,129].

Tanabe et al. treated oro-antral fistulas (OAFs) and oro-nasal fistulas of large size or associated with malignancy using a double flap technique, which involves primary closure with a hinged flap and secondary closure with an insular palatal flap.

Although this surgical approach provides a safer closure, it is quite invasive and requires a longer recovery period [80,130].

The following techniques are considered traditional and commonly used when there is no sinus infection.

#### 4.2.1. Palatal Pedunculated Flap Technique

**Figure 3:**
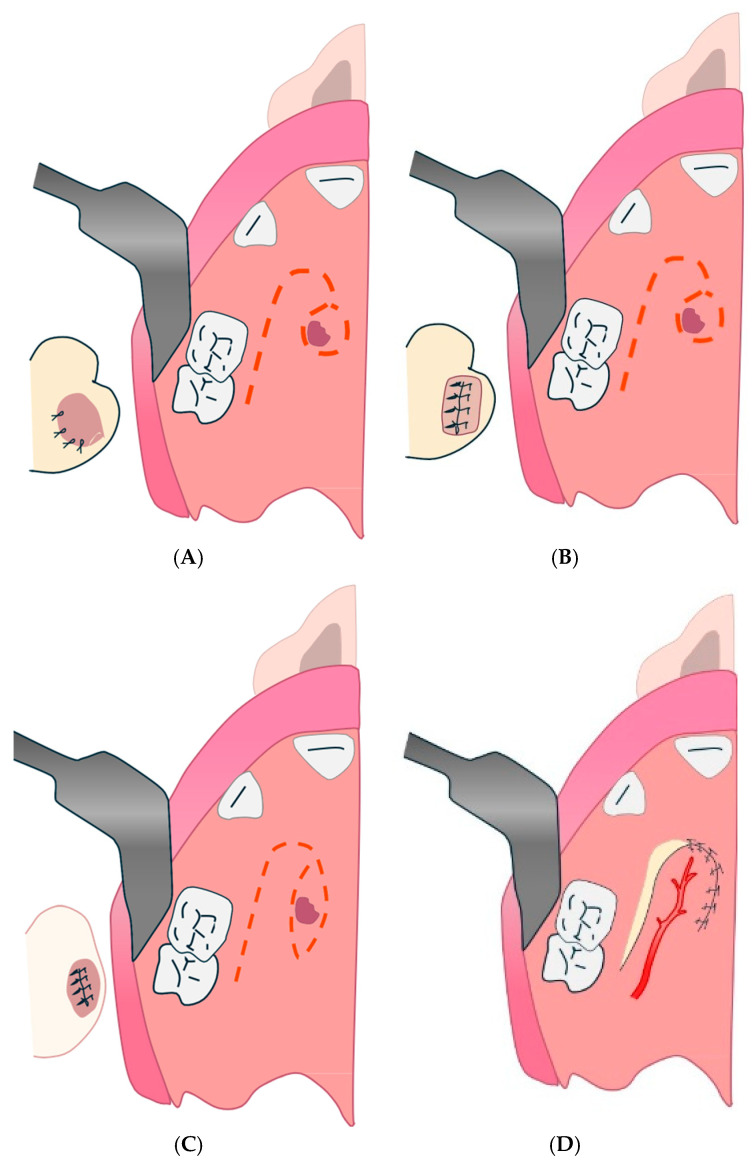
Palatal pedunculated flap. (**A**): Creation of the deep plane by inverting an edge of the perforation. (**B**): Creation of the deep plane by inverting a circular collar around the borehole. (**C**): Creation of the deep plane by inversion of an elliptical mucosal collar. (**D**): Creation of the surface plane by a posterior pedicle palatal flap.

This may be associated with the palatal flap (rotational advancement) described by Gheisari et al., as it is a pedunculated palatine flap used to close small- to medium-sized OAFs (Figure 3) [131].

#### 4.2.2. Cheek Fat Body Flap Technique

**Figure 4:**
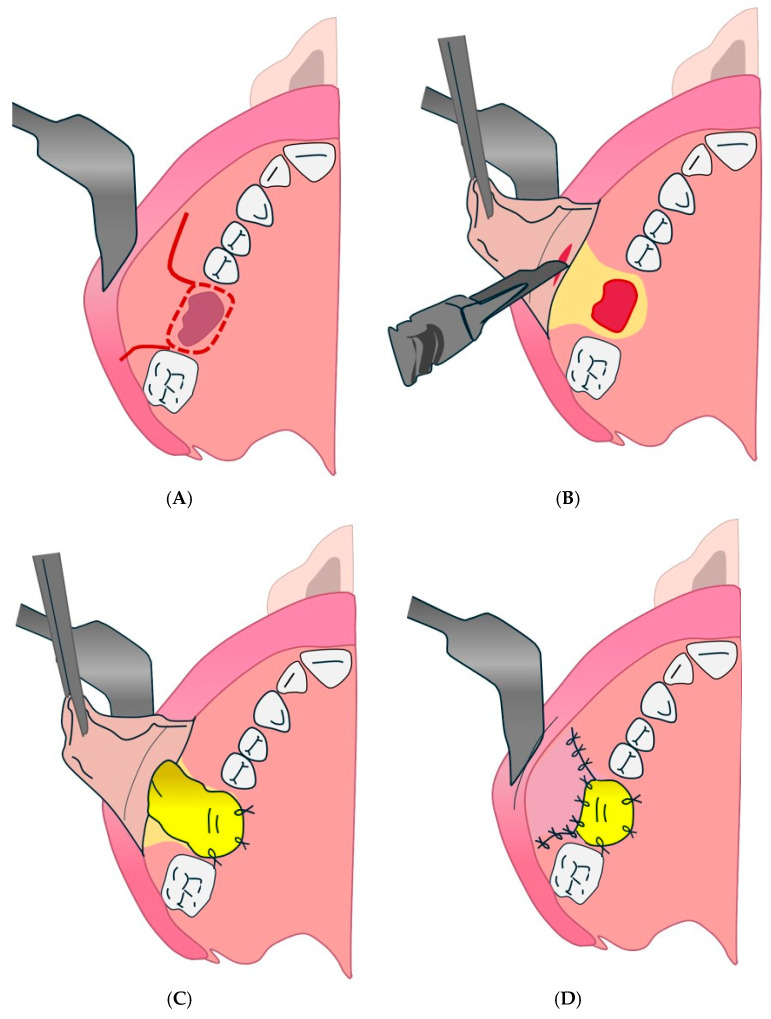
Cheek fat body flap technique. (**A**): Intraoperative view of the oro-antral communication, with exposure of the maxillary sinus cavity. (**B**): Positioning of the mucosal flap and the buccal fat pad (Bichat’s fat pad) to close the communication. (**C**): Final adaptation of the buccal fat pad at the surgical site, ready for suturing. (**D**): Immediate postoperative appearance, with completed closure and tissue stabilization.

**Figure 5:**
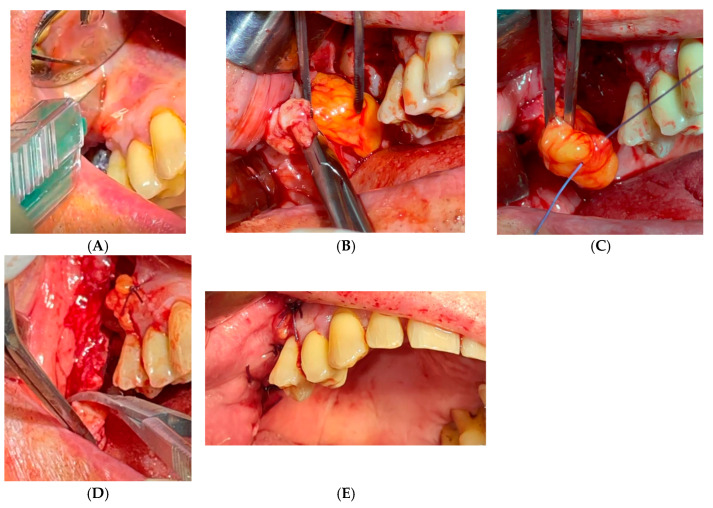
Bichat’s blister flap at the end of surgery. (**A**): scalpel incision. (**B**): Initial presentation of the oro-antral communication site before surgical intervention. (**C**): Preparation and mobilization of the buccal fat pad for placement at the defect site. (**D**): Placement and contouring of the buccal fat pad to fully cover the oro-antral communication. (**E**): Postoperative view showing complete closure and integration of the surgical site.

The cheek fat body flap technique refers to the Bichat flap or “fat bubble flap”, mentioned in several parts of the text (Figure 4 and Figure 5). It is indicated for large OAC or OAF closures (more than 5 mm) and for patients with recurrence or systemic compromise [132].

#### 4.2.3. Posteriorly Pedicled Mono-pedunculated Palatal Flap Technique

**Figure 6:**
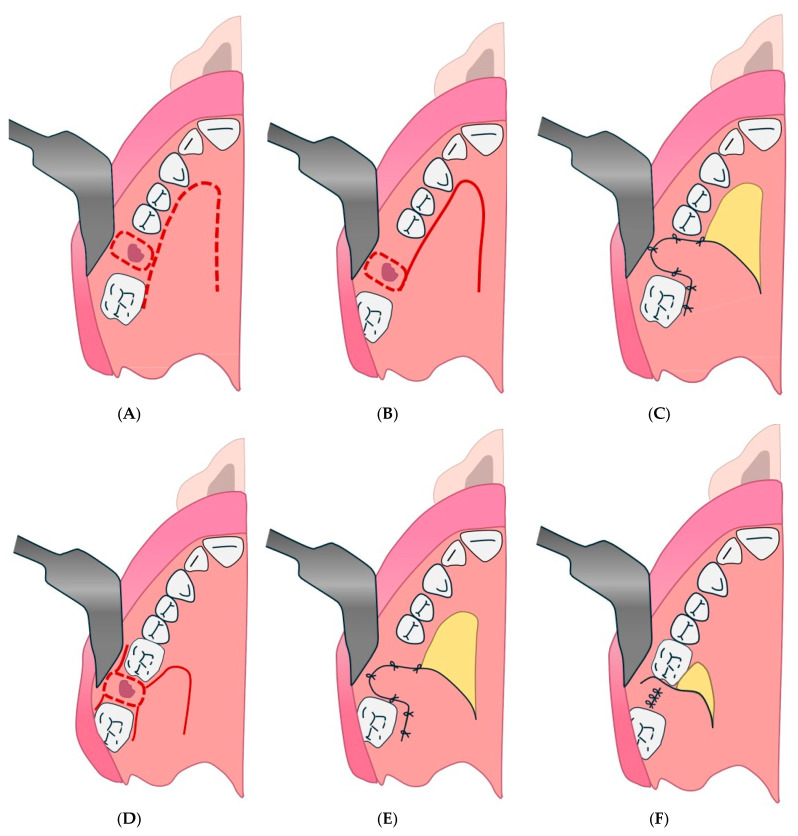
Posteriorly pedicled mono-pedunculated palatal flap. (**A**): Overturning and suturing of one of the edges of the perforation. (**B**) Flipping and suturing of the flap. (**C**): Overturning and suturing of a periorificial collar. (**D**): Flipping and suturing of the flap. (**E**,**F**): Combined plasticity using a posterior pedicled gingival and palatal mono-pedunculated vestibular flap.

The posteriorly pedicled mono-pedunculated palatal flap can be combined with the pedicled palatal periosteal flap described by Blal et al. for OAF closure, which uses a posteriorly pedicled flap to ensure a good success rate and promote tissue regeneration (Figure 6) [77].

#### 4.2.4. Bi-pedunculated Gingivugal Vestibular Flap Technique

**Figure 7:**
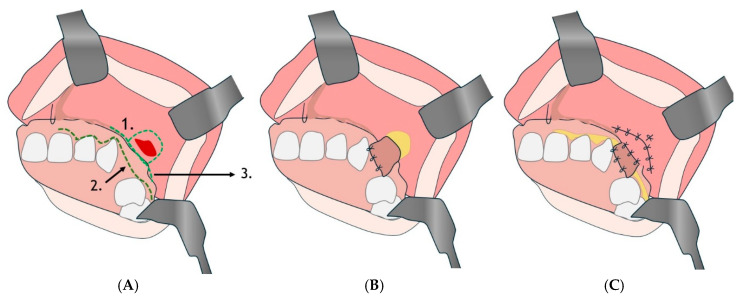
Bi-pedunculated vestibular gingival and jugal flap. (**A**): 1. First inverted U-shaped incision; 2. second incision following the dental collars; 3. third incision parallel to the lower edge of the orifice. (**B**,**C**): Suture flap.

This may correspond to the double flap described in some studies, such as that of Kumar Nilesh, in which a combination of Bichat flap and buccal flap is used for multilayer closure (Figure 7) [133].

#### 4.2.5. Palatal Flap with Posterior Pedicle Technique

**Figure 8:**
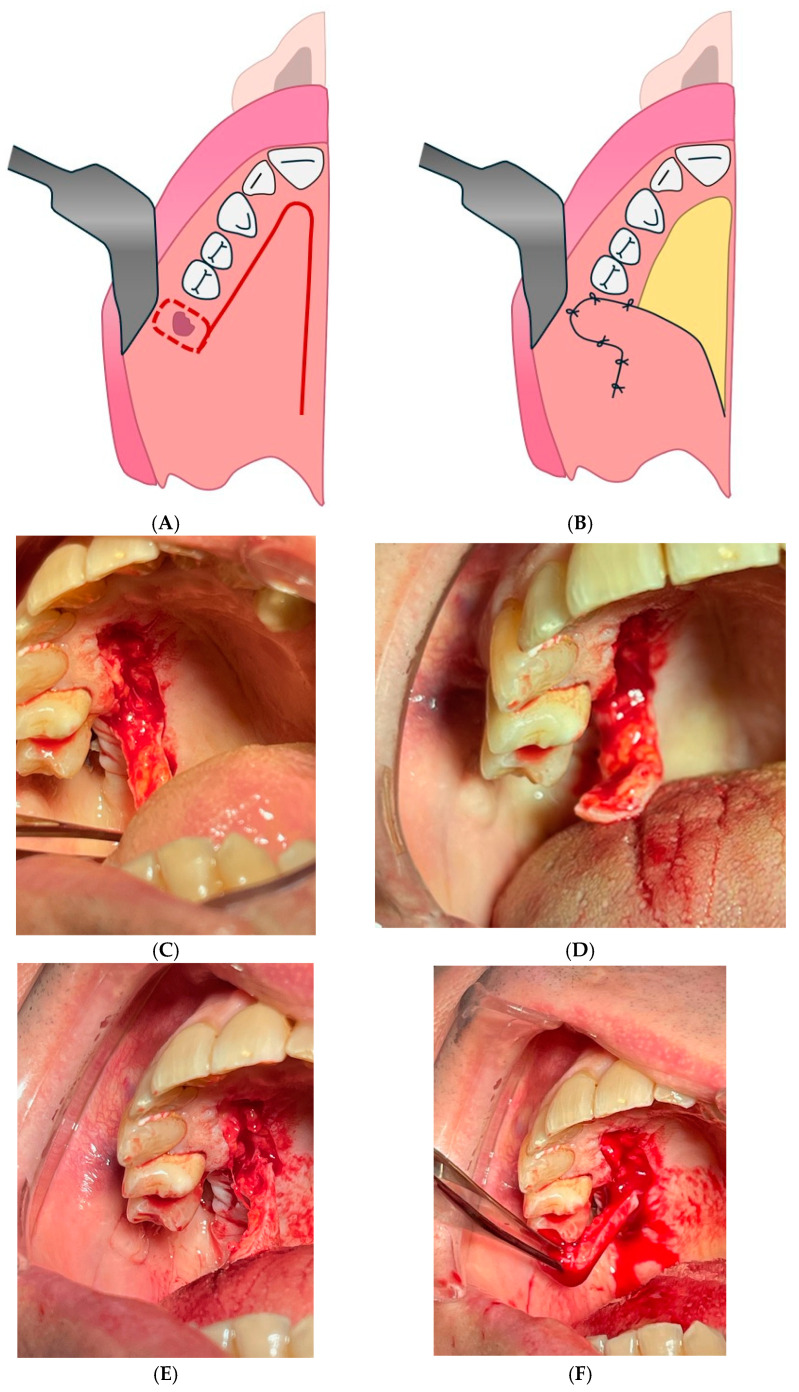
Palatal flap with posterior pedicle. (**A**): flap draw. (**B**): flap suture. (**C**–**F**): clinical surgical passages of inverted palatal flap.

The palatal flap with posterior pedicle technique might coincide with the palatal island flap mentioned by Tanabe et al., which uses a palatine tissue island with posterior pedicle for more complex closures, such as large oro-antral or oro-nasal fistulas (Figure 8) [134].

#### 4.2.6. Plastic for Vestibular Mucosal Slip Technique

**Figure 9:**
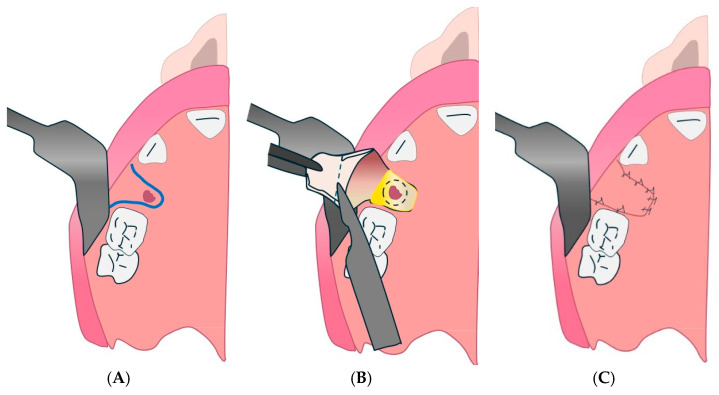
Plastic for vestibular mucosal slip. (**A**). Divergent incisions at the level of the fistula. (**B**). Periosteal incisions. (**C**). Suturing the flap with separate stitches.

In addition, plastic for vestibular mucosal slip technique is particularly useful for medium-sized defects and requires adequate vascularization of the mobilized flap (Figure 9) [135].

Traditional surgical treatment of large OAC or OAF in the absence of sinus infection precludes the placement of local rotational or sliding flaps.

The Bichat flap has the highest success rate and is the most appropriate flap for large OAC or OAF (>5 mm). In addition, the Bichat flap is best suited for the treatment of recurrences, which are particularly common in patients with diabetes, smoking, and cardiovascular disease.

The palatal flap is best suited for the treatment of medium to small OAC or OAF. Such a flap offers a good success rate and is able to promote healing of the gingival tissue.

The buccal flap is the least effective of the other two flaps. For larger defects, it has a high recurrence rate (25%), so it is preferred for medium and small defects.

### 4.3. Large OAC or OAF in the Absence of Sinus Infection—Alternative Techniques

Traditional techniques for treating large OAC or OAF in the absence of sinus infection have shown several limitations, so several authors have conducted studies to introduce alternative treatment techniques.

Kapustecki et al. treated 20 patients with OAC using a combination of autologous bone graft harvested from the mental protuberance or oblique line of the mandible and stabilized with a bi-cortical screw or titanium mini-plate, and a PRF membrane placed over the graft to facilitate its integration. PRF, obtained by centrifugation of blood, is composed of numerous growth factors that promote bone regeneration. The authors found complete OAC closure and alveolar augmentation in all patients and concluded that this therapeutic approach may be a viable alternative to traditional surgical techniques [81,136].

Mahdy Nama et al. compared OAF closure with PRF and 3D mesh versus 3D mesh alone. Although PRF has theoretical advantages, it did not show statistically significant differences from the use of 3D mesh alone. In fact, both techniques were effective with minimal complications [82,137].

Other authors have investigated alternative materials for the treatment of OAC and OAF and concluded that, although further studies with larger numbers of specimens are needed, these techniques are highly effective and offer advantages over traditional approaches.

Ram et al. treated patients with OAF by grafting autologous auricular cartilage harvested from the concal fossa and fixed to the fistulous via in conjunction with a buccal advancement flap. The results of the present study showed that the use of autologous auricular cartilage can ensure the healing of oro-antral fistulas, especially those smaller than 10 mm. Such innovative material, in addition to being biocompatible and easy to harvest, also offers aesthetic advantages; in fact, it leaves no obvious scar at the donor site [83,138].

Jaballah-Magdeleine et al. evaluated the feasibility of using a resorbable porcine-derived collagenous cortical lamina (Lamina^®^ Curve) to close large (>5 mm) OACs. This technique proved to be effective than traditional methods [84,139].

Do et al. conducted a retrospective study of the use of the double-layer technique with collagen–elastin matrix (Matriderm^®^) and polyglycolic acid sheet (Neoveil^®^) to treat oro-antral fistulas (OAF) and oro-nasal fistulas after maxillectomy in patients with oral cancer. The two-layer surgical approach was performed by placing Matriderm^®^ on the surface of the defect followed by Neoveil^®^ fixed with sutures and surgical glue; in some patients, buccal fat was also grafted. Although further studies with larger numbers of specimens and longer follow-up are needed, this alternative technique has shown positive results in reconstructing maxillary defects and reducing the risk of postoperative fistula [85,140].

Hu et al. compared simultaneous OAF closure and sinus floor elevation through a trans-alveolar or lateral approach. The authors concluded that. although the lateral approach is more invasive and results in more postoperative pain and swelling, it provides a greater increase in bone volume [86,141].

The studies reviewed in this section mentioned alternative techniques and innovative materials, summarized below:

- Combination of autologous bone graft and PRF (Kapustecki et al.) [81].
- PRF with 3D mesh (Mahdy Nama et al.) [82].
- Autologous auricular cartilage combined with a buccal advancement flap (Ram et al.) [83].
- Collagenated porcine cortical lamina (Lamina^®^ Curve) (Jaballah-Magdeleine et al.) [84].
- Double layer with collagen–elastin matrix (Matriderm^®^) and polyglycolic acid sheet (Neoveil^®^) (Do et al.) [85].
- Simultaneous OAF closure and sinus floor elevation (Hu et al.) [86].

Alternative techniques are less common or newer, but always used in the absence of sinus infection.

#### 4.3.1. Celesnik Flap

**Figure 10:**
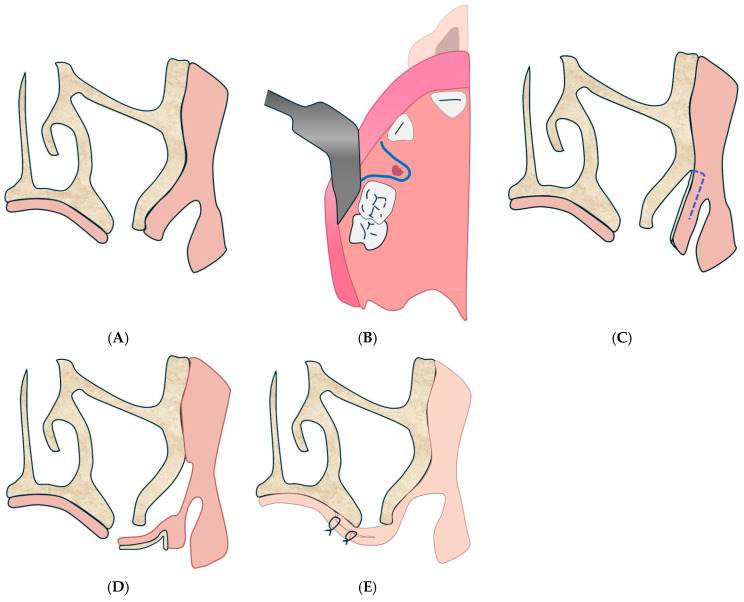
Celesnik flap. (**A**): Preoperative appearance, frontal section. (**B**): Vestibular incision allowing for a very wide externa pedicle mucosal flap. (**C**): Preparation of the gingiva–gingival flap. (**D**): Division of the gingiva-giugal flap and disconnection of the palatal mucosa. (**E**): Suturing of the flap.

Celesnik flap is also referred to as advanced vestibular flap or advanced vestibular mucoperiosteal flap (Figure 10) [142].

#### 4.3.2. Posteriorly Hinged Single-Pedunculated Jugal Flap

**Figure 11:**
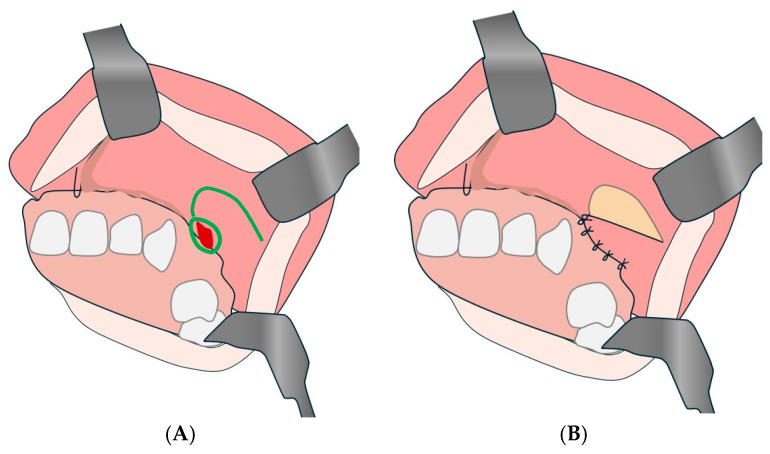
Posteriorly hinged single-pedunculated jugal flap. (**A**): Flap drawing. (**B**): Flap suture.

Posteriorly hinged single-pedunculated jugal flap (Figure 11) [143].

#### 4.3.3. Palatal Flap with Posterior Pedicle

**Figure 12:**
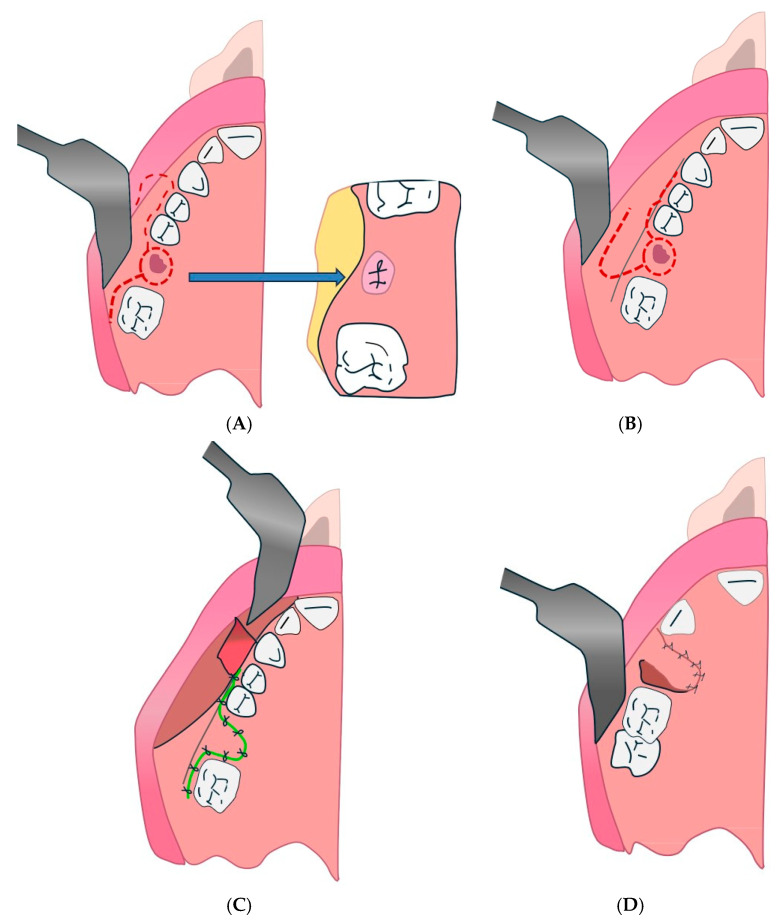
Gingivo-jugal vestibular flap. (**A**): Realization of the deep plane. (**B**): Incision ending in a U shape distally. (**C**,**D**): Realization of sutures without tension.

Another surgical option for treating large or recurrent OAC is the use of the gingivo-jugal vestibular flap (Figure 12) [144], a procedure that combines a gingival flap of the vestibular area with a jugal flap. This technique is particularly useful in cases where the defect is large or difficult to treat with conventional methods. The gingivo-jugal vestibular flap allows for good vascularization and provides stable closure by mobilizing a well-vascularized flap that can cover large defects. The technique involves divergent incisions at the level of the fistula, periosteal incisions for flap detachment, and suturing with separate stitches to ensure tension-free closure.

Although further studies are needed, the following techniques for the treatment of OAC or OAF in the absence of sinus infection may be more advantageous alternatives to traditional approaches.

The combination of autologous bone graft and a PRF membrane provides complete closure of an OAC and alveolar augmentation in all patients.

Treatment of OAF with PRF and 3D mesh versus 3D mesh alone was effective in both cases with minimal complications.

The use of autologous auricular cartilage combined with a buccal advancement flap ensures resolution of OAF, especially when less than 10 mm. This material is biocompatible, easy to transplant, and leaves no obvious scar at the donor site.

The technique of closing large OACs with a resorbable porcine adhesive cortical lamina (Lamina^®^ Curve) is effective and results in less scarring than traditional methods.

The two-layer surgical approach with collagen–elastin matrix (Matriderm^®^) and polyglycolic acid sheet (Neoveil^®^) was effective in reconstructing maxillary defects and reducing the risk of postoperative fistula.

Simultaneous closure of the OAF and sinus floor elevation through a lateral approach provides a greater increase in bone volume than sinus floor elevation through a trans-alveolar approach. The disadvantage of the lateral approach is that it is more invasive and causes more postoperative pain and swelling.

### 4.4. OAC or OAF in the Presence of Sinusitis

In the case of OAC or OAF associated with sinus infection (sinusitis), it is necessary to treat both pathological conditions; several authors have focused their studies on identifying the most effective techniques.

In the case of complicated OAFs, such as those associated with infection, a combined approach of treatment of the infection and closure of the fistula is required, as well as early intervention to prevent more serious complications, such as fungal sinusitis [87,145].

Mishra et al. treated patients with chronic rhinosinusitis caused by OAF with non-surgical therapy via local decongestants and antibiotics for two weeks. Such treatment was effective for fistulas less than 12 mm in size, while more severe cases required combination with surgical therapy with good results [88].

Most authors have concluded that a multidisciplinary and collaborative approach involving professionals, particularly dentists, otolaryngologists, and maxillofacial surgeons, is essential. Sabatino et al. conducted a retrospective study to evaluate patients with OAC and sinusitis treated with various surgical techniques, including FESS and mini-Caldwell–Luc. Within 30 to 90 days, all patients demonstrated complete closure of the communication and resolution of the sinusitis, highlighting the importance of a personalized and multidisciplinary surgical approach [89,146].

Adams et al. confirmed the need for a combined multidisciplinary approach to optimize the treatment of patients with OAF and chronic sinusitis. All patients were treated with FESS and surgical buccal advancement flap or Bichat flap; specifically, buccal advancement flap was used for defects smaller than 1 cm, while Bichat flap was used for defects larger than 1 cm. The endoscopic surgical phase included exploration of the involved sinuses and removal of the necessary sinus and nasal tissues to achieve osteo-meatal drainage [6,147,148]. This combined approach resulted in a 95.5 percent success rate in resolving chronic sinusitis and closing the OAF; there were no statistically significant differences between use of the buccal advancement flap and use of the Bichat flap [149,150].

Gâta et al. further emphasized the importance of a collaborative approach between dentists, otolaryngologists, and maxillofacial surgeons. In fact, the authors compared the management of unilateral odontogenic sinusitis (ODS) with dental treatment and endoscopic sinus surgery (ESS) with or without OAF closure. The results showed that dental treatment had a low failure rate, while ESS associated with OAF closure accelerated healing compared to when it was not associated with closure [69,151].

De Corso et al. conducted a single-center retrospective study and found a 96.5% success rate in treating patients with sinonasal complications of dental treatment with endoscopic sinus surgery and limited sinus mucosal resection. All patients were treated with a multidisciplinary approach involving dentists, otolaryngologists, and radiologists, which optimized outcomes. Specifically, endoscopic surgery included removal of pathologic tissue and creation of an ostium to improve sinus drainage and was combined with dental treatment when possible [152,153,154]. Simultaneous treatment of sinusitis and dental issues ensured rapid resolution of symptoms and significant improvement in patients’ quality of life [90].

Horowitz et al. conducted a study on the treatment of patients with large OAF and chronic sinus disease using a combined endoscopic and Bichat flap approach. They found a high success rate with complete closure of the OAF and minimal complications [91,155].

The Caldwell–Luc technique, historically used to treat chronic sinusitis, has been replaced by other surgical techniques because it was too invasive and not always effective.

Nashef et al. evaluated the modified Caldwell–Luc technique, which is less invasive than the original technique because it does not involve antrostomy of the inferior meatus, to determine whether it was effective in treating patients with odontogenic sinusitis. The results showed that the modified Caldwell–Luc technique can treat most cases of odontogenic sinusitis and reduce the need for subsequent procedures such as FESS [103,156,157]. Endoscopic surgery should only be performed in cases of persistent sinus infection or involvement of other sinuses. In addition, the present study also demonstrated the need to address dental issues to increase treatment success [92,158].

Studies mainly describe combined surgical approaches and regenerative techniques rather than traditional flaps. Techniques mentioned include the following:

Buccal advancement flap (vestibular advancement flap): Used for defects < 1 cm, mentioned by Adams et al. and associated with good results.

- Bichat flap (buccal adipose flap): Used for defects >1 cm, especially in combination with FESS.
- FESS (Functional Endoscopic Sinus Surgery): Endoscopic approach to treat associated sinusitis.
- Mini-Caldwell–Luc: Modified less invasive technique to treat odontogenic sinusitis, mentioned by Nashef et al. [92]
- Combined approaches: Include FESS + Bichat flap or buccal advancement flap.

**Figure 13:**
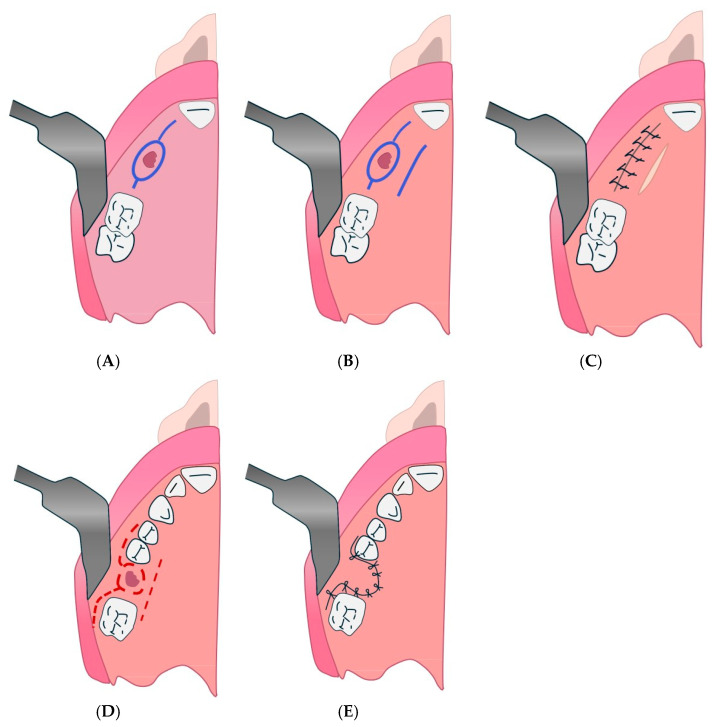
Plastic for palatal fibro-mucosa slip. (**A**): Elliptical engraving around the oro-sinus communication. (**B**): Second palatal incision. (**C**–**E**): Plastic combined with a vestibular mucosal slip and suture.

In the presence of sinusitis, techniques that reduce the risk of contamination and allow effective closure are preferred:

1. Cheek fat body flap technique (Bichat fat pad flap).
2. Plastic for palatal fibro-mucosa slip (Figure 13) [47].

For OAC or OAF associated with sinusitis, a combined approach of treating both conditions as well as early intervention is necessary. Non-surgical treatment with local decongestants and antibiotics for patients with chronic rhinosinusitis caused by OAF is effective only for fistulas less than 12 mm. The management of patients with OAF and chronic sinusitis involves the use of FESS and surgical buccal advancement flaps or Bichat flap; specifically, the buccal advancement flap has been used for defects smaller than 1 cm, while the Bichat flap has been used for defects larger than 1 cm. This combined approach ensures a high success rate and minimal complications. Compared to the original technique, the modified Caldwell–Luc technique (Mini-Caldwell–Luc) is less invasive in treating patients with odontogenic sinusitis and reduces the need for subsequent endoscopic surgery.

Our study has several limitations that should be acknowledged. First, the included studies demonstrated significant heterogeneity in terms of design, sample size, follow-up duration, and surgical techniques employed. This variability introduces challenges in synthesizing the findings and limits the generalizability of our conclusions to all clinical contexts. Additionally, the small number of included studies and their diverse characteristics restricted our ability to perform quantitative analyses, such as meta-analyses or subgroup analyses, which could have provided more robust and statistically grounded insights. Furthermore, our literature search was limited to studies published in English within the last 10 years, which, while ensuring the currency and relevance of the included research, may have excluded older studies or those published in other languages that could have provided additional perspectives or evidence. Finally, the qualitative nature of our synthesis, necessitated by the heterogeneity of the data, prevents definitive conclusions regarding the effectiveness of specific techniques. Future studies with standardized methodologies, larger and more diverse patient cohorts, and longer follow-up durations are necessary to validate and expand upon these findings. This acknowledgment of limitations provides a framework for interpreting our results cautiously and highlights areas for future research. A key limitation of the included studies is the predominant focus on short-term outcomes, with limited data available on long-term success rates and recurrence rates. This reflects a broader gap in existing literature, where practical constraints such as follow-up feasibility or retrospective study designs often preclude extended observation periods. While the short-term outcomes provide valuable insights into the immediate efficacy and safety of various interventions, they may not fully capture the durability of these treatments or the potential for long-term complications and recurrence. Future research should prioritize long-term follow-up studies to assess the sustainability of treatment outcomes over time. Such studies would provide critical evidence to better understand the durability of interventions and inform clinical guidelines for managing oro-antral communication and fistula more effectively.

## 5. Conclusions

Defect size, epithelialization, and the existence of sinus infections all affect how OAC and OAF are treated. Small OACs (less than 5 mm) frequently heal on their own or with conservative treatment that includes hemostatic agents and prophylactics. Surgical closure is usually necessary for larger defects (>5 mm). Because of its strong vascularization, traditional techniques, like the Bichat fat pad flap, are still quite effective for large lesions, whereas palatal or buccal advancement flaps work well for smaller defects. By promoting tissue regeneration and defect closure, novel approaches such as autologous bone grafts, PRF membranes, and resorbable collagen laminae hold promise for complicated patients.

A multidisciplinary approach is essential in sinusitis patients. Effective drainage and closure are ensured when FESS and intra-oral methods are used, leading to high success rates in clearing infections and avoiding recurrence. Additionally, the trend toward less intrusive treatments reflects therapy advances that provide patients with shorter recovery times and lower rates of morbidity. In addition to patient adherence to medicinal therapies and postoperative care, successful management necessitates prompt diagnosis, accurate planning, and cooperation among dental, maxillofacial, and otolaryngology professionals. Nonetheless, the existing corpus of evidence has several drawbacks. The findings may not be as broadly applicable, because many studies are retrospective, have small sample numbers, or lack long-term follow-up. The cost-efficiency and accessibility of modern materials are still little understood, and the relative usefulness of traditional vs emergent procedures is not yet clearly established.

High-quality, multicenter, prospective studies should be the main emphasis of future research in order to confirm the efficacy of innovative materials and methods. Additionally, more research is required to determine the best management practices for patient populations, like those with complicated anatomical differences or substantial comorbidities. The advancement of the profession will depend on the creation of standardized procedures for evaluating results, including metrics reported by patients. By filling in these gaps, more sophisticated and empirically supported methods of managing OAC and OAF will be possible, guaranteeing long-lasting and successful outcomes for a range of clinical situations.

## Figures and Tables

**Table 1:** Featured research in the qualitative analysis and their characteristics.

Authors (Year)	Study Design	Aim	Material and Methods	Outcomes
D.L. Bereczki-Temistocle et al., 2022 [75]	Comparative study	To compare various surgical techniques used to close several OAFs.	Between 2013 and 2020, the medical records of patients with OAF who were hospitalized and treated at the Oral and Maxillo-facial Clinic Targu Mures were examined. General information on reported causes, related illnesses, surgical techniques employed upon admission, and relapses was included in the database.	All large defects (0.6–1.5 cm) treated with buccal advancement flaps relapsed.
Gheisari R. et al., 2019 [76]	Retrospective study	The effectiveness of three distinct surgical approaches for OAF repair is assessed.	Patient records from OAF repair treatments were obtained and examined. Patients’ age, gender, etiology, size, location, duration, and repair procedure were all documented. Patients were split into three groups based on the surgical method utilized to repair the OAF: buccal flap, palatal flap, and buccal fat pad. Local anesthesia was administered to each subject using 2% lidocaine and either 1/100,000 or 1/80,000 epinephrine.	Ostiomeatal complex abnormalities and oro-antral communication had no significant impact on FESS necessity.
K Blal et al., 2020 [77]	Prospective study	Another option for treating OAF is the pedicled palatal periosteal flap, a straightforward and successful surgical procedure with excellent predictability and patient satisfaction ratings.	20 OAF patients underwent closure using a trapezoid flap technique. Closure was immediate in 19 cases, with one small fistula (0.5 mm) resolving within four weeks. Pain peaked in week one (mean 5.5), decreased by week two (mean 2.5), and resolved by week four. Patient satisfaction averaged 9.85.	On a scale of 0 to 10, with 10 denoting complete satisfaction, the mean level of pleasure was 9.85. Another option for treating OAF is the pedicled palatal periosteal flap, a straightforward and successful surgical procedure with excellent predictability and patient satisfaction ratings.
K Nilesh et al., 2020 [78]	Prospective Randomized Clinical Study	The purpose of this study was to evaluate the effectiveness of utilizing a buccal fat pad and buccal mucosa advancement flap to close the oro-antral communication in two layers.	Patients with oro-antral communication were divided into two groups at random; group B received single layer closure (buccal fat pad) and group A received two layer closure (buccal fat pad and buccal mucosa). Both groups underwent the same perioperative care procedure. The length of the procedure, pain, swelling, mouth opening, and surgical success (full closure without any nasal regurgitation) were among the postoperative criteria assessed.	On 7 and 30 postoperative days, respectively, the two groups’ postoperative assessments of mouth opening and discomfort did not reveal any statistically significant differences. However, using a combination of the buccal advancement flap and the buccal fat pad resulted in comparatively greater swelling.
Channar et al., 2021 [79]	Comparative cross-sectional study	To evaluate the differences between buccal advancement flap and double layer closure in the treatment of OAF.	Participants were randomly divided into two groups: Group I received treatment with a buccal advancement flap, while Group II underwent “double-layer closure” using a buccal fat pad and a second layer with a buccal advancement flap.	Any postoperative problems, such as wound dehiscence, necrosis, or infection, were assessed.
Tanabe et al., 2024 [80]	Comparative study	The purpose of the study was to present the closure method for oro-antral or oronasal fistulas utilizing palatal island flaps with or without hinge flaps. We described the surgical procedures, evaluated their effectiveness, and contrasted them with other closure techniques.	This study included nine patients with oro-antral or oronasal fistulas treated between 2000 and 2022. Closure techniques involved either a single flap or a double flap (palatal island and hinge flaps). Causes included trauma, cysts, and tumors. Five cases used double flap closure, while four used single flap closure.	Following treatment, none of the nine patients had significant postoperative problems or flap necrosis.
M Kapustecki et al., 2016 [81]	Comparative study	This study aimed to evaluate the value of PRF and autogenous bone graft in promoting normal bone regeneration at the oro-antral communications location.	Twenty individuals had their bone regeneration at the oro-antral communication point evaluated. Autogenous bone grafts from the oblique line in six cases and the mental protuberance in fourteen cases were used to augment bone deficiencies. A PRF membrane was placed over the graft.	In the study group in all cases closure of the oro-antral connection was observed. The alveolar’s average height was 12.5 mm, and its average width was 13 mm. Three patients experienced an average 1.5 mm rise in alveolar height.
Nama and Ghanim, 2022 [82]	Prospective study	This study aims to evaluate the impact on patients with persistent oro-antral fistulas of surgical repair utilizing PRF with customized 3D printed mesh.	16 patients with chronic oro-antral fistulas, aged 16–68, were enrolled in this study. They received surgical repair at AL-Wasity Teaching Hospital from March 2020 to August 2021 using PRF with customized 3D printed mesh.	The highest proportion of the study patients in both the PRF and mesh groups were within the age group (<60) years (87.5% and 62.5%, respectively.) Regarding fistula size, 50% of patients in the PRF group and 75% of patients in the mesh group had fistulas that were less than 20 mm in size, and 50% of patients in the mesh group showed healing, compared to 37.5% of the total.
Ram H. et al., 2016 [83]	A Prospective Clinical Study	Our current work concentrated on a novel method for closing OAF that uses a buccal advancement flap to support an autogenous auricular cartilage graft.	Twenty patients with OAF were included in the study. Following the removal of the fistular tract, a buccal mucoperiosteal flap and auricular cartilage were placed over the defect to create a double layer closure. The posterior auricular technique was used to harvest the transplant. Patients were assessed after one week, three weeks, six weeks, and three months.	We discovered that an autogenous auricular cartilage transplant works well as a sealing agent for the closure of OAF. Because it makes sinus lifting simple, we suggest this method for defects smaller than 10 mm2 where future dental implant implantation is desired.
Jaballah-Magdeleine et al., 2024 [84]	Retrospective study	This study sought to evaluate the effectiveness of utilizing collagenated porcine cortical lamina to repair OACs greater than 5 mm.	This study included 34 cases of OAC larger than 5 mm who underwent surgical repair using a porcine-derived collagenated cortico-cancellous plate, (Lamina Curve^®^). The median patient age was 46 years. The study cohort consisted of 12 females and 22 males. The median follow-up time was 54 days. complete mucosa assessed the 4th week.	During the fourth week after surgery, the main result was the presence of full mucosal closure. Adverse events and stitching disunion were secondary results.
Do et al., 2024 [85]	retrospective single center experience	This study examines the efficacy of a novel double-layer technique that uses Matriderm^®^ and Neoveil^®^ to close oro-antral and oronasal fistulas (OA/ONFs). Neoveil^®^ is a biodegradable mesh sheet that acts as a barrier to stop leaks and scarring, while Matriderm^®^, an acellular dermal matrix made of collagen and elastin fibers, promotes tissue regeneration.	Twelve patients who had maxillectomy surgery for oral cancer between January 2022 and May 2023 were the subjects of retrospective research. Analysis was carried out on patient data, such as defect size, bone invasion, and tumor stage. R software was used for statistical analysis to assess the results of the surgical procedures, which included sinus mucosa preservation and either buccal fat grafting in conjunction with the double layer technique or the double layer approach alone.	With lower T stages, no bone invasion, smaller defect dimensions, and intact sinus mucosa all associated with lower fistula risk (*p* < 0.05), the findings show a 41.7% incidence rate of fistula formation. Interestingly, no patients needed surgical corrections for fistulas, confirming the method’s effectiveness.
Hu et al., 2023 [86]	Comparative study	The purpose of this study was to examine two innovative methods for elevating the maxillary sinus floor and close chronic OAFs.	Between January 2016 and June 2021, ten patients with persistent OAF undergoing implant placement were studied. OAF closure and sinus floor elevation were performed, and postoperative outcomes were compared using *t*-test and χ^2^ test.	The findings show a 41.7% incidence rate of fistula formation with intact sin, smaller defect diameters, lower T stages, and no bone invasion. Neither group experienced any serious issues.
Verma and Verma, 2022 [87]	Observational study	To examine three different techniques for elevating the maxillary sinus floor and close chronic OAFs.	We report three cases of OAF, managed by three different techniques. We also suggest a combined approach for large OAFs, repaired in 3 layers using septal cartilage, fat, and a buccal muco-periosteal advancement flap.	We also suggest a combined approach for large OAFs, repaired in 3 layers using septal cartilage, fat, and a buccal muco-periosteal advancement flap.
Mishra et al., 2016 [88]	Observational study	A non-surgical method of treating rhinosinusitis linked to a persistent oro-antral fistula from tooth extraction was assessed.	Methods: A total of 26 consecutive patients, 15 men and 11 women, with ages ranging from 28 to 72 years (mean age of 49.81 years), received antibiotics for 10 days and local decongestion therapy for 2 weeks. After two weeks, patients exhibiting a decline in Sino-Nasal.	Results of Test 22 after two weeks of continuation. Those who did not show any progress had surgery, while those with test scores of 22 after two weeks continued weekly local decongestion therapy for up to six weeks. Findings: At two
Sabatino et al., 2023 [89]	Retrospective study	This study aims to demonstrate our experience in appropriately managing individuals with odontogenic sinusitis who have oro-antral communication and fistula.	Techniques: 41 patients with a diagnosis of odontogenic sinusitis with oro-antral communication and fistula were included in this retrospective study based on the inclusion criteria; one patient had pre-implantological difficulties, fourteen had implantological complications, and 26 had classical complications.	Results: thirteen patients received solely oral treatment, twenty-six patients received a combination, and two patients had fractioned combined therapy. All the enrolled patients experienced a full remission of their symptoms and a closure of their fistula.
Adamska et al., 2024 [26]	Retrospective Study	This study set out to assess the effectiveness of a combination surgical technique for oro-antral communications repair.	The efficacy of combining buccal advancement flap or buccal fat pad grafting with functional endoscopic sinus surgery was assessed in this retrospective study. Following a combination of functional endoscopic sinus surgery and oro-antral communication closure using a buccal advancement flap or buccal fat pad graft for persistent oro-antral communication, 43 patients with chronic sinusitis from 2007 to 2013 were assessed.	Preoperative computed tomography, antibiotic therapy, sinus pathologic tissue exploration and removal, rotation of a pedicled fat pad graft into the oral opening, oral mucosa repair and closure, sinus and nasal tissue excision required for osteomeatal drainage establishment, and follow-up to assess treatment success or failure were all part of the treatment.
Anda Gâta et al., 2021 [69]	Comparative study	To compare the outcomes of dental therapy and endoscopic sinus surgery for odontogenic sinusitis (ODS).	Medical records of ODS patients were analyzed. Group one had ODS from periapical pathology treated with dental care or FESS, with treatment success measured by resolution rates. Group two had ODS with chronic oro-antral communication. Kaplan–Meier curves compared healing times for patients undergoing combined FESS versus OAF closure alone using the Log-Rank test.	Of the 45 patients in group one, 25 received only dental care, while 20 chose to have only FESS treatment.
De Corso E. et al., 2022 [90]	Retrospective study	Our goal was to present our actual experience treating patients with the endoscopic endonasal technique, including information on symptoms, etiology, disease progression, and success rate.	This study reviewed 262 cases (2015–2022), with 44.65% dental and 55.34% iatrogenic causes. Patients underwent customized endoscopic treatment, with combined dental care in 58% of cases. Quality of life improved significantly within 15 days of post-surgery.	There was a notable increase in quality of life 15 days following surgery.
Horowitz et al., 2016 [91]	Comparative study	To explain a one-stage combined peroral buccal fat pad flap and endoscopic sinus surgical technique for big OAF producing chronic maxillary sinusitis.	This study reviewed OAF patients with chronic rhinosinusitis treated from 2010–2013. Exclusions included malignancy, recurrent fistulas, radiation history, or age < 18. Surgery involved flap closure and endoscopic sinus drainage. Outcomes assessed were OAF closure, complications, and revision surgery needs.	Following the initial treatment, the paranasal sinuses on the treated side fully recovered, and all 44 patients (97.8%) had their sinuses closed. The group’s follow-up period was 7.6 ± 4.3 months on average (7–21 months).
Nashef et al., 2024 [92]	Retrospective study	By using a modified Caldwell–Luc technique, which involves entering the maxillary sinus through the canine fossa without making a counter-opening in the inferior nasal meatus, the study aims to quantify the frequency of retreatment of maxillary sinusitis of odontogenic origin after treatment.	A total of 82 patients with odontogenic sinusitis treated surgically using the modified Caldwell–Luc technique at the Department of Oral and Maxillofacial Surgery, Poriya Medical Center, between 2014 and 2021 were included in this retrospective cohort analysis. Nonodontogenic sinusitis patients were not included.	Retreatment with FESS is needed if maxillary sinusitis symptoms persist for over four weeks after a modified Caldwell–Luc procedure despite proper medical care.

## Data Availability

Not applicable.

## Abbreviations

CBCT	cone beam computed tomography
OAC	oral-antral communication
FESS	Functional Endoscopic Sinus Surgery
OAF	oro-antral fistula
ODS	odontogenic sinusitis
PRF	platelet-rich fibrin
